# Draft Genome Sequences of Six *Rhodobacter capsulatus* Strains, YW1, YW2, B6, Y262, R121, and DE442

**DOI:** 10.1128/genomeA.00050-14

**Published:** 2014-02-13

**Authors:** Hao Ding, Michelle M. Moksa, Martin Hirst, J. Thomas Beatty

**Affiliations:** a Department of Microbiology and Immunology, University of British Columbia, Vancouver, Canada; b Centre for High-Throughput Biology, University of British Columbia, Vancouver, Canada

## Abstract

*Rhodobacter capsulatus* is a model organism for studying a novel type of horizontal gene transfer mediated by a phage-like gene transfer agent (RcGTA). Here we report the draft genome sequences of six *R. capsulatus* strains that exhibit different RcGTA properties, including RcGTA overproducers, RcGTA nonproducers, and/or RcGTA nonreceivers.

## GENOME ANNOUNCEMENT

The purple nonsulfur photosynthetic alphaproteobacterium *Rhodobacter capsulatus* is a model organism for studying a novel mechanism of horizontal gene transfer mediated by gene transfer agents (GTAs), bacteriophage-like entities whose sole function is to mediate gene transfer (1). The production of GTAs appears to be regulated in response to environmental signals. The study of the regulation of GTA production has been carried out mainly on the RcGTA of *R. capsulatus* strain B10 (2) and its derivative SB1003, the only genome-sequenced strain of *R. capsulatus* (3), and other poorly characterized B10-derived RcGTA overproducer strains, Y262, R121, and DE442, generated by chemical mutagenesis (4, 5). However, the molecular mechanisms responsible for these RcGTA overproducer strains remain unknown. In addition, some *R. capsulatus* environmental isolates display altered phenotypes in RcGTA production and/or reception (6). To extend our understanding of RcGTA biology, we sequenced the genomes of the *R. capsulatus* strains YW1, YW2, B6, Y262, R121, and DE442 (Table 1).

**TABLE 1:** Related properties of *R. capsulatus* strains

Strain	Isolation source (reference)	GTA production^*a*^	GTA reception^*a*^	Avg coverage^*b*^	No. of contigs^*c*^	Accession no.	Version
YW1	Yellowwood State Forest, IN (6)	No	No	158×	54	AYPY00000000	AYPY01000000
YW2	Yellowwood State Forest, IN (6)	No	Yes	178×	65	AYPZ00000000	AYPZ01000000
B6	St. Louis, MO (6)	No	Yes	156×	116	AYQA00000000	AYQA01000000
Y262	Chemical mutagenesis of BB103, a spontaneous streptomycin-resistant derivative of B10 (4)	Overproducer	Yes	233×	54	AYQB00000000	AYQB01000000
R121	Derived from Y262, *crtG* mutant (5)	Overproducer	Yes	212×	39	AYQC00000000	AYQC01000000
DE442	Believed to be derived from Y262, *crtD* mutant (B. Marrs, personal communication)	Overproducer	Yes	198×	40	AYPR00000000	AYPR01000000

a GTA production and reception data are based on experiments done under laboratory conditions as previously described (12).

b Average coverage refers to k-mer coverage.

c There are strings of Ns within contig sequences representing the estimated gap length based on the paired-end read information.

Genomic DNA from each *R. capsulatus* strain was isolated from a late-log-phase culture grown in the minimal medium RCV (7), using the 2012 JGI bacterial DNA isolation CTAB protocol (http://my.jgi.doe.gov/general/protocols/JGI-Bacterial-DNA-isolation-CTAB-Protocol-2012.pdf). DNA concentrations were quantified using the Qubit dsDNA HS Assay kit with the Qubit 2.0 fluorometer (Life Technologies). A total of 2 µg of each DNA sample was used to build indexed PCR-free libraries for each genome using a TruSeq kit (Illumina Hayward, CA). Sequencing was performed on an Illumina MiSeq platform using indexed paired-end 250-nucleotide v2 chemistry. Reads were aligned with the SB1003 reference genome using the Illumina MiSeq Reporter v 2.2 using BWA (8), and assemblies were performed using the MiSeq Reporter v2.3.32 running Velvet (9, 10). The genome sequences of strains Y262, R121, and DE442 were assembled using a k-mer length of 150 with SB1003 as the reference genome (chromosome and plasmid concatenated; NCBI reference sequences NC_014034.1 and NC_014035.1) for contig ordering, whereas the natural isolates YW1, YW2, and B6 were assembled using a k-mer length of 200 without a reference genome. Average k-mer coverages of 158× (YW1), 178× (YW2), 156× (B6), 233× (Y262), 212× (R121), and 198× (DE442) were achieved. Genome annotation used the NCBI Prokaryotic Genome Annotation Pipeline (version 2.0).

Our results show that the poorly provenanced *R. capsulatus* strains Y262, R121, and DE442 are closely related to strain SB1003, because of conserved within-contig syntenies. In addition, 95.2% (Y262), 93.8% (R121), and 94% (DE442) of sequence reads were aligned to the SB1003 genome. Contigs from strains DE442 and R121 lack genes from the SB1003 plasmid pRCB133, confirming results of a DNA microarray study (11). The environmental isolates exhibit relatively diverse genome composition, with only 80.5% (YW1), 76.1% (YW2), and 74% (B6) of the reads mapping to the SB1003 genome. Although none of the three environmental isolates appear to produce RcGTA, they all contain RcGTA structural gene clusters.

### Nucleotide sequence accession numbers.

This whole-genome shotgun project has been deposited in DDBJ/EMBL/GenBank. The accession number and version of each genome sequence described in this paper are listed in Table 1.
